# Negativland - a home for all findings in psychology

**DOI:** 10.1186/2050-7283-1-2

**Published:** 2013-02-27

**Authors:** Keith R Laws

**Affiliations:** Department of Psychology, School of Life and Medical Sciences, University of Hertfordshire, Hatfield, AL10 9AB UK

## Abstract

Psychology has been historically plagued by the under-reporting of both replications and null findings. The avoidance of these core ingredients of scientific practice means that the psychology literature is unquestionably distorted. The bias in psychology is pervasive and systemic, afflicting researchers, reviewers, editors and journals, all of whom are wed to pursuing the novel and the curious at the expense of the reliable. Psychology therefore operates in a manner that is askew of other sciences, with the links between replicability and believability seemingly much weaker. Additional problems follow from the distorted way that psychology currently operates - including spinning findings, publication bias, and sadly, outright fraud. Such problems represent a serious challenge for psychologists to get their house-in-order – and one step is to make sure that replications and null findings find a home in psychology rather than remain our dirty little secrets that further eat away at the credibility of our science.

*“…if the goal of scientific research is to render established truths, then the neglect of replication must be viewed as scientific irresponsibility”* (Smith, [1], p.971).

“But Professor Laws, our findings are negative and so we don’t have much to write about*”* Anyone who has taught a psychology lab will have heard a variant of this phrase innumerable times. The corollary is usually a discussion containing the obligatory reference to “…our results would/could/should be significant if we had tested more participants”. This aversion to the null hypothesis (or so-called *negative findings*) is by no means new to psychology, and “…*is arguably one of the most pernicious and unscientific aspects of modern social science*.” (Fergusson & Heene [2], p.4). Most ominously though, psychology seems to fare worse than many other disciplines, has done for a long time, and has done little to rectify this situation.

Psychological knowledge is not acquired *a priori* - we cannot know in advance what will emerge as reliable findings without replicating initial findings. Nevertheless, as Hartshorne & Schachner [3] note “…replicability is not systematically considered in measuring paper, researcher, and journal quality. As a result, the *current incentive structure rewards the publication of non-replicable findings*” (p3 my italics). Indeed, psychologists have received criticism for being overly-fixated on the kudos attached to the pursuit of novelty; and the publishing of highly counter-intuitive, sometimes career-making controversial papers in high-impact journals - which are themselves symbiotically wed to pursuing the glamorous and the curious. Like any science, psychology has few identifiable destinations, but many routes and many cul-de-sacs and as a creative enterprise, psychologists will produce unexpected and even seemingly untenable results. Part of the scientific process is unquestionably to generate new knowledge, models and change our ‘world view’, but in doing so, to accurately delineate reliable from unreliable findings partly through the analysis of their replicability.

Humans are, however, inclined to seek out information that confirms rather than falsifies or refutes their beliefs. Contrary to Sir Karl Popper’s passionate advocacy of *falsification*, *confirmation bias* is the all-too-human tendency to seek results that confirm our pre-existing beliefs and knowledge systems. Ironically, most of what we know about confirmation bias comes from psychology, yet our discipline is amongst the worst offenders in this regard - with psychologists tending to more favourably rate results that conform to their prior expectations than people in other disciplines (Hergovich et al. [4]). Moreover, this represents no real change from earlier analyses of the same phenomenon in psychologists as far back as the 1970s (Goodstein & Brazis, [5]) – psychology tells us psychologists are *confirmist conformists* - preferring to seek *positive* evidence in favour of their pet theories.

Despite the negativity about ‘negativity’, null results are as fundamental to the advancement of science as attention-grabbing positive results - whether one has a Popperian view of science or not. Likewise, replications are fundamental to science – even leading psychologists to propose the somewhat impractical notion that all experiments should be replicated before publication (Lykken, [6]). The debate over both null findings and replications is thrown into relief with evidence of publication bias (against negative results) being regularly documented in meta-analyses. Given the human capacity for generating ideas and an exponentially increasing number of scientific papers published, the claim by John Ioannidis [7] claim that “most published research findings are false” seems quite plausible. Negative findings and replications are science’s road signs telling us how to moderate our journey – we may like to ignore them or find them frustrating, but they are vital to progress - and contrary to existing trends, journals must allocate more space and importance to both null findings and replications.

## Acceptance and rejection

*The moral of this story is that the finding of statistical significance is perhaps the least important attribute of a good experiment; it is never a sufficient condition for concluding that a theory has been corroborated, that a useful empirical fact has been established with reasonable confidence—or that an experimental report ought to be published.* Lykken ([6] p 158)

For a paper to be published, or even submitted, depends largely on how the results fare against the ‘ontological mystique’ of the *p* <.05 level (Rosnow & Rosenthal, [8]). Naturally, many results do not pass the ‘interocular traumatic test’ (Edwards, Lindman & Savage, [9]) or, in plain terms, hit you right between the eyes. But as Edwards et al. say “the enthusiast's interocular trauma may be the skeptic's random error… [and] A little arithmetic to verify the extent of the trauma can yield great peace of mind for little cost” (p.217).

The *inference revolution* that took place in psychology between 1940 and 1955 led to inferential statistics and *p* values becoming *de rigueur* (Gigerenzer & Murray, [10]). The percentage of articles published in American Psychological Association (APA) journals that used statistical tests rose from 17% in the 1920s to almost 90% by the 1960s; and although near ceiling, has continued to rise: 91.7% for the 1970s, 92.6% for the 1980s, and 93.9% for 1990 to 1998 (Hubbard & Ryann, [11]). As soon as significance testing appeared, however, criticism began (see Boring [12]), has continued and led some even to describe statistical testing as psychology’s “dirty little secret” (Lambdin [13]).

Nevertheless, one upside of being tied to the p<.05 cut-off is that we can readily estimate if expected numbers of papers are attaining and missing this benchmark. Examining 165 articles in four major APA experimental psychology journals published between 1986–7, Sterling et al. [14] reported that while most (94.3%) used significance tests, crucially the vast majority *rejected* the null hypothesis (93.5%). Moreover, not much had changed from Sterling’s comparable analysis in 1959, where 97.3% of papers published in four major psychology journals reported statistically significant outcomes for their major hypotheses. By contrast, although 94.3% of psychology articles used significance tests in 1986–7, only 69.2% of articles in medical journals did so and while most rejected the null hypothesis, it was not as often as in psychology journals (95.6 vs. 85.4%). So, psychologists not only more commonly use statistical tests than medics, but more frequently reject the null hypothesis and have been consistently doing so for some time.

### Bias – a systemic problem in Psychology

*“This violent bias of classical procedures [against the null hypothesis] is not an unmitigated disaster. Many null hypotheses tested by classical procedures are scientifically preposterous, not worthy of a moment's credence even as approximations. If a hypothesis is preposterous to start with, no amount of bias against it can be too great. On the other hand, if it is preposterous to start with, why test it*?” Edwards (1965, p 402)

The bias against publishing negative findings in psychology means that they remain unknown to other investigators. Worse though is the likelihood that negative findings are independently replicated and each is unpublished, until eventually by chance - one study obtains a *spurious* significant effect and is published. Like a moth to a star, such findings attract attention – especially since such false positives are also quite likely to be counter-intuitive and unusual. By contrast, when researchers do manage to publish null findings, they typically arrive with little or no fanfare and at a much slower pace than positive outcomes (Ioannidis et al. [15]). Hence, negative findings are not only less common, but when they are published their impact on the literature is weakened.

But where is the bias occurring? The answer is - at multiple levels. Some psychology journals and editors even express a policy of not publishing replications regardless of whether the outcome is positive or negative. This was thrown into relief by the furore surrounding Daryl Bem’s ‘Feeling the Future’ paper (2011) purporting to show precognitive abilities. Some may wonder how a paper making such claims could be published in a high impact mainstream psychology journal. I do not, however, share this concern since the paper was peer-reviewed and deemed appropriate by the editor. Nor do I subscribe to the somewhat *anti-science* view of Edwards (1965) quoted above, that some hypotheses are too preposterous to test. For me, the importance of Bem’s paper has nothing to do with whether we *believe* in his demonstration of psychic ability, but how such studies perform an important self-assessment checking function for psychologists. As the authors (Ritchie, Wiseman & French [16]) of a replication attempt state, this study raises issues about “…*our current statistical paradigm, the policies of academic journal publishing, and what exactly a scientist needs to do to convince the world that a surprising finding is true*”. Two salutary lessons from this episode are: first, that no matter how many times one author documents something in a paper (and Bem had 9 experiments), the work requires independent replication; and second, the difficulty researchers typically experience when trying to publish replications. As noted, some journals do not publish replications, including that which published the Bem study. Although Ritchie et als ‘failed’ replication (and we need a better descriptor than this) found a good home, it may not have or like many other non-replications – hidden away in a less well read and lower impact journal, which is a travesty– remarkable though false findings inhabiting palaces, while the truth resides in ghettos. Entirely consistent with such notions, we know that highly cited papers are less likely to replicate (Ioannidis, [7]) and that publication bias seems to affect high-impact journals more severely (Ioannidis, [7]; Munafò et al., [17]).

We might wonder if psychologists view replications differently from scientists in other disciplines? Francis [18] has contentiously argued that for psychologists, the link between replication and belief in a phenomenon is not as strongly correlated as in other sciences. Francis highlights the Bem [19] study as an example of one where the paper contains multiple replications of a phenomenon and yet we don’t believe it, while other phenomena do not so readily replicate (e.g. bystander apathy) but we do believe in them! Though of course, the former is replication within the same lab as opposed to replications across labs – and this is a key difference. Nonetheless, Francis bleakly concludes that “*The scientific method is supposed to be able to reveal truths about the world, and the reliability of empirical findings is supposed to be the final arbiter of science; but this method does not seem to work in experimental psychology as it is currently practiced*.”

Strong editorial bias undeniably exists against publishing direct replications. In a survey of 79 editors of social science journals, 94% indicated that replication studies were not encouraged in the editorial policy (Neuliep & Crandall [20] p. 87). Seventy-two per cent preferred to publish studies claiming novel findings rather than replications. In a parallel survey of *reviewers* for social science journals (Neuliep & Crandall, [21]), 54% stated a preference for new findings than replications, arguing that the latter were “Not newsworthy” or even a “Waste of space”. Despite the biased attitude of editors and reviewers for social science journals, the situation is somewhat different in the natural sciences. Madden, Easley, and Dunn [22] compared the attitude toward replications between editors of journals from the social and the natural sciences. Whereas the comments of the editors from the social sciences journals are similar to the ones reported by Neuliep and Crandall [20] the natural science journal editors present a more varied though more positive picture, with comments ranging from “Replication without some novelty is not accepted” to “Replication is rarely an issue for us . . . since we publish them.”

To complete a triptych alongside the bias of editors and reviewers, we must add researchers themselves. While an associate editor of the *Journal of Personality and Social Psychology (JPSP)* during a 3-month period, Greenwald [23] conducted a survey of reviewers and authors. Remarkably, Greenwald found that researchers were *eight times* more likely to submit a manuscript for publication if the results were positive rather than negative. In a similar survey Coursol and Wagner [24] sent questionnaires to 1000 APA members involved in psychological therapeutic outcome research. Of 609 respondents, the decision to submit a paper for publication was *significantly related to the outcome*. When studies had positive outcomes (i.e. clients improved) 82% submitted their paper, but with negative outcomes (client did not improve) only 43% submitted their articles. Furthermore, of studies reporting a positive outcome 66% were published, while only 22% of the neutral/negative outcome studies were published i.e. leaving 78% unpublished! (for similar conclusions from a recent meta analysis, see Hopewell et al. [25]).

Despite the bias, more replications probably do exist in the literature than is commonly believed. Researchers tend to notice only *exact* replications, but most are in fact partial or conceptual replications i.e., where researchers typically ‘tweak’ the methods of previous studies (Neuliep & Crandall [26]). In the most recent analysis of this issue, Makel, Plucker & Hegerty [27] examined 500 randomly selected articles from the top 100 psychology journals since 1900 - to find that just 1.6% of publications were replications. Of these, only 14% were direct replications that follow the original 'experimental recipes’, while the vast majority were conceptual replications that test related hypotheses using different methods and settings

Even when replicating, many authors refrain from even using the word ‘replication’ in a title possibly through fear of having their paper rejected (because they are not being sufficiently original) Indeed, the word ‘replication’ appears in the titles of psychology articles now as little as it did 15 years ago (<50 times per year), although it does appear three times as often in psychology abstracts (though again the number is not increasing) – suggesting some reticence on the part of researchers to ‘out’ their replications.

### Flash-bang-wallop science: Two scientists are racing for the sake of all mankind

Although the origins and consequences of publication bias have been debated over decades, this bias continues to *increase*. Pautasso [28] examined the phrase ‘non-significant difference’ in abstracts over 40 years and found a decrease through time in the ratio of non-significant-to-significant results reported in the natural, medical and social sciences. Fanelli [29] examined the actual outcome (rather than the abstract) in over 4600 publications from different countries and disciplines and found strong evidence for a steady and significant increase in publication bias across time. The frequency of papers declaring significant statistical support for their *a priori* formulated hypotheses increased by 22% between 1990 and 2007 alone. Crucially, however, psychology and psychiatry were the worst offenders (amongst 19 disciplines) being especially prone to the bias of publishing positive findings – being *five times* more likely to report a positive result than space sciences (which were at the other end of the spectrum).

The attraction to the novel is underscored by increased use of the somewhat grand phrase ‘*paradigm shift’* in article titles. This phrase, derived from Thomas Kuhn’s *The Structure of Scientific Revolutions*[30] directly or indirectly implies a seismic change in how we view some major phenomenon. In this context, Atkin [31] noted the use of *paradigm shift* in *titles* pointing out how it had increased to 30 instances per year by 2001. A quick analysis of the subsequent period 2002–2012 by myself revealed over 1400 instances and a clear year-on-year increase (with 77 in 2003 and 198 in 2011). Although most instances occur in medicine, psychology has over 70 article titles containing this grandiose phrase in the past 9 years – in Kuhnian terms, it is unlikely that psychology has experienced one paradigm shift nevermind 70 recently. In conjunction with the reduction of null findings and simple replications, it seems that scientists are *driven* to make grandiose claims about their own work.

With the ever-increasing numbers of academic papers published annually, many more psychologists now actively promote their papers to hopefully raise them above the colossal mass. The promotion of science should be a key venture of scientists, however, it does raise the spectre of *spin*. Yavchitz et al. [32] examined spin in scientific papers and press releases. From 498 press releases in the *EurekAlert* database (December 2009 to March 2010) they extracted all two-arm, parallel-group RCTs (*n* = 70) for analysis. Defining ‘spin’ as *“…specific reporting strategies (intentional or unintentional) emphasizing the beneficial effect of the experimental treatment*”, they identified spin in 40% of article abstract conclusions and in 47% of press releases. Examining the moderating effect of: journal type, funding source, sample size, type of treatment (drug or other), results of the primary outcomes, press release author and the presence of ‘spin’ in the abstract, the only factor associated with “spin” in the press release was “spin” in the article abstract. Furthermore, 21 (51%) of the associated news stories were reported with ‘spin’ mainly the same as that as identified in the press release and article abstract conclusions. Although some scientists may find that journalists misrepresent their findings, some scientists wilfully participate in what we might euphemistically call the over-zealous promotion of their work (or themselves).

Alongside career-based factors, a publish or perish culture and the growing allure of media profiles for scientists, the bias in psychology against null findings and replications not only bias the literature, but may lead to a small minority of psychologists to engage in questionable practices - and several recent high profile cases accord with this impression. Fanelli [33] found that 1.97% of scientists admitted to having fabricated, falsified or modified data or results at least once and up to 33.7% admitted other questionable research practices. In surveys asking about the behaviour of colleagues, rates were 14.12% for falsification, and up to 72% for other questionable research practices. Turning specifically to psychologists, John, Lowenstein & Prelec [34] surveyed ‘questionable research practices’ (QRPs) in almost 6000 academic American psychologists. The researchers asked the questions using a method that attempts to make people more honest, in part by giving them an incentive to tell the truth. They found that a surprising number of psychologists had engaged in questionable research practices. Based on the anonymous replies from 2155, the majority were guilty of selectively reporting studies that ‘worked’ (67%), failing to report all dependent measures (74%), continuing to collect data to reach a significant result (71%), reporting unexpected findings as expected (54%: so-called *HARKing* – see below), and excluding data post-hoc (58%). Approximately 35% indicated that they had doubts about the integrity of their own research on at least one occasion. And 1.7% admitted to having faked their data. Interestingly, the highest rates of QRPs emerged for those working in cognitive, neuroscience, and social disciplines, and among researchers using behavioural, experimental, and lab-based methodologies. John et al. conclude that “*the prevalence of QRPs raises questions about the credibility of research findings and threatens research integrity by producing unrealistically elegant results that may be difficult to match without engaging in such practices oneself. This can lead to a “race to the bottom,” with questionable research begetting even more questionable research*.” (p.8). In this respect, Questionable Research Practices may be the academic equivalent of ‘performance enhancers’, used by those who feel they need that ‘something extra’ to compete with high performing competitors.

### Meta-analysis, the Grey Literature and the File Drawer problem

With the rising tide of studies being published, we look increasingly to meta-analyses to quantitatively summarise literatures for us. Unsurprisingly, the number of meta-analyses has increased exponentially, with over 5600 papers the phrase ‘meta-analysis’ in the title published last year (2012).

Meta-analysis is based on quantitatively summarising findings that are accessible in the literature and this largely (though not exclusively) means *published* works. We could, however, try to locate data from unpublished studies – the so-called *Grey Literature.* Again, however, it seems that psychology may not fare so well. A Franco-Dutch study (Schöpfel et al. [35], Farace et al. [36]) analysed 64 *scientometric* articles published between 1987 and 2005, citing several thousands references, to estimate the proportion of grey literature cited in different disciplines. While engineering sciences had 39–42%, Education 14–19%, biology 5–13; however, at the bottom of the barrel again, we find psychology with 3% and Psychiatry with 1%.

Nonetheless, the grey literature may introduce its own biases. Unpublished studies located by authors are likely to reflect a small sub-sample of all unpublished studies given the difficulties with tracking down such information currently e.g. with unregistered trials. Although Dickersin et al. [37] reported that the grey literature may be more a result of failure to write up results rather than the rejection of submitted manuscripts, doubts about quality prevail. An analysis of 60 meta-analyses that included published and unpublished trials found that unpublished trials were less likely to conceal intervention allocation adequately and to blind outcome assessments (Egger [38]). Unpublished and published research also differs because investigators halt studies at preliminary stages when data do not favour the experimental treatment (Dickersin, Chan, Chalmers, Sacks, & Smith, [37]). Unpublished research is more likely to have small samples, which may reflect pilot projects, difficult-to recruit subjects, or highly innovative interventions. In a similar fashion, Lipsey and Wilson [39], in a meta-analysis of 92 meta-analyses of outcome research in the areas of psychotherapy and education, found that the average effect size was 0.53 and 0.39 for published and unpublished research, respectively (for examples from drug efficacy, see Hart, Lundh and Bero 2012). Some discrepancy exists between the willingness of authors and editors to include grey literature. For example, Cook et al. [40] assessed attitudes toward inclusion of unpublished data in meta-analyses and found a clear majority in favour amongst the authors of 150 meta-analyses (78%), but interestingly journal editors were far less convinced (47%).

Ferguson and Brannick [2] examined 91 meta-analyses published in *American Psychological Association* and *Association for Psychological Science* journals for the methods used to identify and control for publication bias. Of the 91 studies, 70% made some effort to analyze publication bias, and 41% reported finding evidence of bias. In an effort to control publication bias, 63% of studies attempted to find unpublished studies. The authors conclude that “…many meta-analyses in psychology exhibit both asymmetric funnel plots and small enough summary effects to appear fragile in the presence of publication bias” (p.126). Surprisingly, however, those meta-analyses that included unpublished studies were just as likely to find evidence for publication bias as those that did not. Authors of meta-analyses were themselves overrepresented in unpublished studies acquired, leading. Ferguson and Brannick to argue that searches for unpublished studies may actually *increase* rather than decrease some sources of bias (see also Rothstein and Bushman [41]; Fergusson & Heene, [2]).

In contrast to narrative reviews, meta analysis both focuses on and attempts to estimate publication bias. In 1979, Rosenthal argued that “The extreme view of the "file drawer problem" is that journals are filled with the 5% of the studies that show Type I errors, while the file drawers are filled with the 95% of the studies that show nonsignificant results” (p.638). Although Ioannidis (2006) proposed that “most published findings are false”, we should not ignore the possibility that *many unpublished findings are true*. Nobody knows how many negative studies are tucked-away in a file-drawer; however, statistical techniques derived for use with meta-analysis allow us to estimate the file-drawer effect (for a clear overview of the various techniques for assessing publication bias, see Møller & Jennions, [42]).

Despite fears about missing unpublished studies, techniques aligned to meta analysis do now permit an estimate of the number of missing effect sizes, their direction and even how large those missing effect might be. The standard way to look for bias in a meta-analysis is to examine funnel plots of the individual study effect sizes plotted against for example, their sample sizes or the standard errors. When no bias exists, studies with larger samples and smaller error will cluster around the mean effect size. By contrast, smaller samples and greater error variance produce far more variable effect sizes (in the tails). Ideally, we should observe a nicely symmetrical inverted funnel shape, with smaller studies producing greater variability in effect size outcomes.

Crucially, we can impute missing values to see how it changes the overall effect size in a meta-analysis. This method, known as *trim and fill* was devised by Duval and Tweedie [43] and is based on adding studies to a funnel plot until it becomes symmetrical. Smaller studies are omitted until the funnel plot is symmetrical (trimming) and the trimmed funnel plot is used to estimate the true “centre” of the funnel; and then the omitted studies and their missing “counterparts” around the centre are replaced (filling). This provides a more accurate estimate for an adjusted effect size that includes the “filled” studies. A recent study that used the trim and fill method to analyse 48 meta-analyses, from the Cochrane Database of Systematic Reviews, estimated that 56% had at least one study missing whereas the number of missing studies in 10 was statistically significant and in four reviews, would lead to significant changes in the conclusions (3 that were significant became nonsignificant and one that was nonsignificant became significant).

## Power, null findings and meta-analysis

*Among the many virtues that have been extolled for meta-analysis, the main appeal is that it can convert existing things into something better. “Significance” can be attained statistically when small group sizes are pooled into big ones (Feinstein, [*[44]*], p. 71).*

Statistical power refers to the probability that the test will reject the null hypothesis when the null hypothesis is false (or in other words, the probability of not making a Type II error). Underpowered studies, waste resources as they lack the power needed to reject the null hypothesis. While underpowered studies fail to detect genuine effects, the converse – in an overpowered study, essentially trivial effects may become significant. So, we skate somewhere between profligacy and triviality (and sometimes blindly as initial power calculations are often not performed). Given the high rates of rejecting the null hypothesis in psychology (Sterling [14]), this profusion of positive outcomes is even more striking given the size of effect detected and the fact that studies are often underpowered). In this context, Rossi [45] calculated power for 6,155 statistical tests in 221 journal articles published in the 1982 volumes of the *Journal of Abnormal Psychology, Journal of Consulting and Clinical Psychology*, and *Journal of Personality and Social Psychology*. The power to detect small, medium, and large effects was .17, .57, and .83, respectively. The average statistical power for medium effect sizes had hardly varied from the estimate of .48 given by Cohen (1962) almost 30 years earlier (see also Sedelmeir and Gigerenzer [46]). This worrying scenario is emphasised by Schmidt and Hunter [47] who reported that with “*0.50 as a rough average …This level of accuracy is so low that it could be achieved just by flipping a (unbiased) coin!*” (p. 40). Unsurprisingly, this had led some to have serious reservations about the published literature and especially about the potentially artefactual nature of some controversial findings (Rossi [48]). The cocktail of low power, small effects and a high rejection of the null hypothesis, undoubtedly means many findings will be unreliable.

Of course the lack of power in studies inevitably leads to speculation that larger studies/ trials are required to establish an effect. Decisions about whether additional studies are warranted is often a moot point especially since a majority (e.g. streptokinase: Lau et al. [49]) or even *all* negative (null) findings in a meta-analysis may produce an overall significant effect size. A recent example an overall significant effect emerges in meta-analysis despite each individual study being nonsignificant would be the use of LSD to treat alcoholism (Krebs & Johansen, [50]). Egger and colleagues have written extensively on the unreliability of conclusions in meta-analyses where small numbers of nonsignificant trials are pooled to produce significant effects [51].

## Losing our religion

One thing we have learned from meta-analysis is that effect sizes often diminish with time – the purported *decline effect* – leading some to even suggest the equivalent of the Heisenberg Principle at play (with observers no longer being naïve when observing the phenomenon under investigation: Schooler, [52]). One reason is that study-quality or experimental techniques e.g. neuroimaging typically refine and improve over time. A classic example concerns the initial CT studies reporting greater ventricle brain ratios in people with schizophrenia; however, the difference shrank and shrank until regression analysis was predicting that ventricle-brain ratios would soon be larger in controls than patients (van Horn &McManus, [53]). This shrinking effect size is a pervasive effect – it occurs whether one is looking at modern brain imaging techniques, medicine, psychological therapy or regular experimental psychology studies.

Hyped ‘major’ findings emerge initially with large effects and small samples (as we have seen), only to shrink as time progresses. One problem is that meta-analytic validation of an effect “is not seen as necessary to proclaim an effect reliable. Textbooks, press reports, and narrative reviews often rest conclusions on *single influential articles rather than insisting on a replication across independent labs and multiple contexts*” (Giner-Sorolla [54], p 564, my italics).

## Conclusion

Issues relating to replicability combined with recent examples of blatant fraud have re-focused the already hovering spotlight on the credibility of psychological science. Pashler & Wagemnakers [55] recently remarked that psychology is perhaps finding itself to be “…*the public face for the replicability problems of science in the early 21st century, [and] psychological science has the opportunity to rise to the occasion and provide leadership in finding better ways to overcome bias and error in science generally*.” (p.529). This leadership will however require psychologists to take a more active role in submitting replications and null findings – science is clearly not self-correcting. (Pashler & Harris [56]).

Although some individuals are culpable, many of the problems in psychology are systemic. Psychology may be fortunate (or unfortunate) to have uncovered so few rogues and as in any sphere of life, some will always exist. Increased vigilance needs to be married with systemic changes and although we cannot rectify every issue immediately, we may begin to address some, including the problem of ‘negative’ results. Although some specialist replication and null journals have appeared, they have either not lasted very long (e.g. in the late 70s, the journal *Replications in Social Psychology* stopped after three volumes) or have continued and with modest outputs (*Journal of Articles in Support of the Null Hypothesis* has published just over 30 papers in over 10 years). Although laudable, such journals create a *special* space for replications and null findings rather than acknowledging their place in the centre of science. Turning to our approach at *BMC Psychology*, it is journal policy to publish work- deemed by peer reviewers “…*to be a coherent and sound addition to scientific knowledge and to put less emphasis on interest levels, provided that the research constitutes a useful contribution to the field*.” This remit unquestionably includes the consideration of null results and replications and the more central roles they need to play in the discipline. Journal editors have previously tried to provide directives to encourage replications, but this alone may even be counter-productive. For example, Evanschitzky et al. [57] reported that the drive to publish replications in marketing journals had actually led to a 50% *reduction* in replications published. We must acknowledge that while providing a home for replications and null findings, researchers must change their mentality….as they say, the ball is in your court!

Studies return negative results for many reasons including a lack of statistical power and thus researchers should routinely report effect sizes to evaluate this possibility. As we have seen, publication bias distorts meta-analytic reviews especially where the probability of locating studies reflects the *strength* and *direction* of the findings– which may be extensive in some cases. Statistical adjustments are helpful, but they too depend upon the selection of published studies made by the analyst, which itself may misrepresent the available published data. Searching the *grey literature* is also helpful, but again has its own biases. Neither statistical estimations of bias nor trawling unpublished studies ultimately addresses the underlying issue – most negative findings remain unpublished but more need to be so.

The systemic cultural bias exhibited by editors, reviewers and authors against negative findings undoubtedly has a further subtle, but pernicious outcome when authors do publish negative findings. Some simply downplay or fail to mention their negative findings, overuse phrases such as ‘nonsignificant trend’ or worse, may try to reframe as negative results as positive. Some may avoid having totally negative results through what has become known as *HARKing* (Kerr, [58]): *Hypothesizing After the Results are Known*. This is where researchers represent their hypotheses as coming prior to results, when in fact they came after the results and were aligned to fit. A recent spoof article (though perhaps closer to tragedy than comedy) argued that *a priori* scientific hypothesizing is the most reliable form of precognition because so few psychology papers state hypotheses that turn out to be disconfirmed (Bones, [59]). We cannot avoid the conclusion that psychologists, editors, and reviewers have consistently conspired to deny the existence of negative results and the importance of replication – these are Psychology’s dirty little secrets… that we need to change.
